# Conservation Agriculture Affects Grain and Nutrient Yields of Maize (*Zea Mays* L.) and Can Impact Food and Nutrition Security in Sub-Saharan Africa

**DOI:** 10.3389/fnut.2021.804663

**Published:** 2022-01-26

**Authors:** Yamdeu Joseph Hubert Galani, Ivy S. Ligowe, Martin Kieffer, Donwell Kamalongo, Alfred Mexon Kambwiri, Pamela Kuwali, Christian Thierfelder, Andrew J. Dougill, Yun Yun Gong, Caroline Orfila

**Affiliations:** ^1^School of Food Science and Nutrition, Faculty of Environment, University of Leeds, Leeds, United Kingdom; ^2^Chitedze Agricultural Research Station, Lilongwe, Malawi; ^3^Centre for Plant Sciences, University of Leeds, Leeds, United Kingdom; ^4^Centre for Environmental Policy and Advocacy, Blantyre, Malawi; ^5^Civil Society Agriculture Network, Lilongwe, Malawi; ^6^International Maize and Wheat Improvement Centre, Harare, Zimbabwe; ^7^School of Earth and Environment, Faculty of Environment, University of Leeds, Leeds, United Kingdom

**Keywords:** maize varieties, nutrition security, micronutrient deficiencies, Malawi, climate change

## Abstract

Maize is a major staple and plays an essential role in food and nutrition security in Sub-Saharan Africa (SSA). Conservation agriculture (CA), a climate-smart agriculture practise based on minimum soil disturbance, crop residue retention, and crop diversification, has been widely advocated but without extensive research on the impact it may have on maize nutrient composition, and food and nutrition security. This study assessed the grain yield, macro- and micronutrient mineral content, and nutrient yield of eight maize varieties grown in Malawi, and how these are affected by CA practises over two seasons. The minerals were analysed by inductively coupled plasma (ICP) coupled to optical emission spectroscopy (OES) and to mass spectroscopy (MS). Grain yield and Se content differed among the varieties, while C, N, Fe, K, Mg, Mn, P, and Zn were similar. The local variety *Kanjerenjere* showed lowest grain and nutrient yields. The open-pollinated varieties (OPVs) concentrated more minerals than the F1 hybrids, but the latter showed higher yields for both grain and nutrients. Typical consumption of the eight maize varieties could fully meet the protein and Mg dietary reference intake (DRIs) of Malawian children (1–3 years), as well as Mg and Mn needs of adult women (19–50 years), but their contribution to dietary requirements was low for Fe (39–41%) and K (13–21%). The trials showed that CA increased grain yield (1.2- to 1.8-fold) and Se content (1.1- to 1.7-fold), but that it had no effect on C, K, Mg, P, and Zn, and that N (1.1- to 1.2-fold), Mn (1.1- to 1.8-fold), and Fe (1.3- to 3.4-fold) were reduced. The high increase in grain yield under CA treatments resulted in increased yields of protein and Se, no effect on the yields of K, Mg, Mn, P, Zn, and reduced Fe yield. Conservation agriculture could contribute in reducing the risk of Se deficiency in Malawian women and children but exacerbates the risk of Fe deficiency. A combination of strategies will be needed to mitigate some of the foreseen effects of climate change on agriculture, and food and nutrition security, and improve nutrient intake.

## Introduction

Maize (*Zea mays* L.) is the most produced and third most consumed cereal crop in the world, after wheat and rice (1). Maize is the main staple food crop of more than 300 million Africans, especially in Sub-Saharan Africa (SSA) (2, 3). In Malawi, for example, average maize consumption is 337 g/person/day for adults, which can supply 1,069 kcal/capita/day, thus dominating energy intake (1, 4). Additionally, maize porridge is the principal complementary food given to infants and children as weaning food to meet their energy requirements. It is usually first served as thin porridge and later in infancy, it is substituted by thicker porridge and complemented with soups of vegetables and fish (5, 6). The low nutritional value of complementary foods is associated with a high incidence of stunting in low-income countries (6–8). Therefore, it is essential to understand the factors affecting the nutritional quality of maize to predict and mitigate nutrient deficiencies and related diseases.

Micronutrient deficiencies (MNDs) have been identified as major public health problems affecting a large part of world population, with pregnant women and children under 5 years at the highest risk (9, 10). Between 1.5 and 2 billion people have one or more chronic MNDs, notably in minerals I, Fe, Se, Zn, and vitamins, such as folate and vitamin A; the majority live in Africa and Asia (1, 9, 11–13). In Malawi, for instance, more than 50% of households were estimated to be at risk of Ca, Zn, and/or Se deficiencies (14). The 2015–2016 Malawi Micronutrient Survey showed that Fe deficiency affected 22% of preschool children (6–59 months), 5% of school-aged children (5–14 years), and 15% of non-pregnant women of reproductive age, and that Zn deficiency affected 60% of children and 63% of women (15). Moreover, household-level food consumption data on Malawi estimated that 70% of the population consumed inadequate levels of Se (14), and that deficiency levels of two Se markers were found in 29.6 and 62.5% of women (16).

Micronutrients play important roles in human health, and MNDs can retard growth and cognitive development, impair immunological functioning and increase the risk of non-communicable diseases, such as skeletal, cardiovascular, and metabolic disorders (17, 18). They can also contribute to intellectual impairments, perinatal complications, and increased risk of morbidity and mortality (9). As a result of high prevalence of MNDs, malnutrition indicators in SSA are of concern: in Malawi, stunting affected 37.1% of children under 5 years, underweight 12.8%, and wasting 2.7% (19, 20). Micronutrient deficiencies occur when intake and absorption of vitamins and minerals are too low to sustain good health and development (21). Consequently, the nutritional composition of maize grain is critical in determining micronutrient intakes from the average Malawian diet.

Food composition data are important in nutritional planning and provide data for epidemiological studies. In fact, food consumption or supply data can be used to calculate dietary mineral intakes and infer deficiency risks among populations. Risk estimates are sensitive to the quality of composition data, especially for elements required in small quantities (22). Moreover, the nutrient composition of maize kernels is influenced by many factors, such as genetic background of varieties, plant age, geographic location, and environmental conditions (23). High variability in nutrient contents among maize varieties was reported (24), but there is not enough information on grain nutrient composition of different maize varieties, especially in SSA. Besides, the current trend in maize development is not solely towards high-yielding hybrids and stress-resistant varieties, but also towards developing nutritionally improved varieties (25). To achieve this, it is essential to identify maize varieties delivering improved grain quality for human consumption and industrial processing (26), when cultivated with local and climate-smart farming practises. Therefore, to generate baseline information for the development of more nutritious maize, the nutrient composition of hybrids and open-pollinated varieties (OPVs) of maize landraces needs to be assessed.

Climate change can exacerbate undernutrition through three main pathways, among which household food security, i.e., access to safe, affordable, sufficient, and nutritious food (27). Climate change adaptation strategies with mitigation co-benefits including integrated crop, soil, and water management measures, can be used to reduce soil degradation and increase the resilience of agricultural production systems to the impacts of climate change (28, 29). Conservation agriculture (CA) is a climate-smart farming practise characterised by minimum soil tillage, permanent soil cover with organic materials, and diversification of crop species grown in sequence and/or in association (30). It increases the capacity of agricultural households to adapt to climate change and can lead to increased soil organic carbon over time (28). From 2015 to 2016, CA was practised on about 12.5% of the total global cropland (180 million ha) and its adoption was reported by 78 countries, representing a 69% increase of cropland and 42 more countries since 2008/09 (31).

In SSA, CA in maize farming has been shown to improve yield, maize resistance to drought stress (32), soil structure and properties (33, 34), and dietary diversity and quality (35). In Malawi, the adoption of CA by smallholder farmers is recommended by government policy (36). Nevertheless, the adoption of CA in Africa is lowest in the world, with only 1.51 M ha cropland area in 2015–16, representing only 1.1% of cultivated land on the continent, and 0.8% of global CA cropland area (31). Recent studies from Zambia have shown that together with resilience, the benefits of improved nutrition will make CA more attractive for smallholder farmers (35). However, little efforts have, to date, been placed on potential synergistic benefits of CA on food and nutrition security, and little is known about CA impact on maize nutritional quality. Hence, this study aimed to evaluate the grain yield, mineral content, and nutrient yield of eight maize varieties grown in Malawi, and assess how CA practises influence these parameters under field trial conditions. How these compositional differences could impact food and nutrition security is analysed and discussed.

## Materials and Methods

### Experimental Design

The experiments were carried out at Chitedze Agricultural Research Station (CARS), Malawi, during the 2018–19 maize growing season (December to May), and the CA trial was repeated in the 2019–20 season. Chitedze Agricultural Research Station is located at latitude 13.9738°, longitude 33.6527°, and altitude 1,147 m.a.s.l. The predominant soil texture in this site is sandy loam (66 sand and 25% clay), classified as ferruginous *Latosol* (also known as *Alfisol*). The climate is sub-humid tropical with unimodal rainfall distribution, typically starting in November and ending in March, and annual rainfall typically averages 800–900 mm (32, 34).

For the varietal trial, seeds of the eight white maize varieties (three OPVs and five F1 Hybrids) typically grown in Malawi were used (Table 1). The trial was conducted using a randomised block design with four replicate plots per treatment (variety). Each plot measured 1.5 × 5 m, and plots were separated by 1.5 × 1.5 m free space. The land was prepared according to conventional Malawian maize farming practises. It consisted of traditional 15- to 20-cm high ridges separated by 75-cm furrows, and the maize seeds were sown at an approximate depth of 10 cm on top of the ridges, using a dibble stick, after the first effective rain. Spacing followed the *Sasakawa* global 2,000 maize production practises, 75-cm inter-row and 25-cm intra-row, to achieve a plant population of 53,333 plants/ha. Basal application of NPK(S) + Zn 23:10:5 (6) + 6 fertiliser at 100 kg/ha occurred at sowing, followed by urea (46% N) at 69 kg/ha, 28 days after sowing (DAS). Crops were rain-fed during the 2018–2019 growing season, and the field trial received 793 mm of rainfall. Weeds were controlled using a hand hoe as soon as they appeared, three times during the cropping season. Fall armyworm on the maize crop was controlled using insecticide Chlorpyrifos applied at 1,500 g/ha approximately 6 weeks after sowing.

**Table 1:** Characteristics of the eight maize varieties used in this study.

**Sr. no**.	**Release name**	**Pollination type**	**Seed source**	**Maturity range (days)**	**Suitable agroecologies**	**Additional traits**
1	Local or Traditional (*Kanjerenjere*)	OPV	Local market	Late (150–160)	All agroecological zones	Drought sensitive
2	ZM 309	OPV	Demeter seeds	Very early (90)	Dry mid-altitude	Drought tolerant, flint-, and MSV-resistant
3	ZM 523	OPV	Demeter seeds	Early (110–130)	Dry mid-altitude	Drought tolerant, MSV-resistant
4	DKC 9089	Hybrid	Decalbs seeds	Early to medium (115–120)	Wet and dry mid-altitudes	Medium drought tolerant, MSV-, and GLS-resistant
5	DKC 8053	Hybrid	Decalbs seeds	Medium (120–140)	Wet and dry mid-altitudes	Medium drought tolerant, MSV-, and GLS-resistant
6	MH 30	Hybrid	Demeter seed	Medium (120–140)	Wet and dry mid-altitudes	Good drought tolerant, MSV-, and GLS-resistant
7	PAN 53	Hybrid	Pannar seeds	Medium (120–140)	Wet and dry mid-altitudes	Medium drought tolerant, MSV-, and GLS-resistant
8	SC 719	Hybrid	Seed Co	Late (150–160)	Wet mid-altitude	Medium drought tolerant, MSV-, and GLS-resistant

*OPV, open-pollinated variety; MSV, maize streak virus; GLS, grey leaf spot*.

The CA trial consisted of maize variety DKC 9089, a popular Monsanto/Bayer F1 Hybrid selected for its tolerance to many maize diseases. The trial tested seven combinations of farming practises, including monocropping, intercropping, or crop rotation with grain legumes cowpea (*Vigna unguiculata* L.), pigeon pea (*Cajanus cajan* L.), and velvet bean (*Mucuna pruriens* L.) laid out on plots of 24 × 13.5 m size with 18 rows of maize in a randomised complete block design with four replications and eight treatments as follows:

■ T1 = conventional practise: ridge and furrow system made with hand hoes, continuous maize monocrop with crop residues removed;
■ T2 = CA basin maize: no-tillage system with planting on basins (0.15 × 0.15 × 0.15 m), continuous maize monocrop, crop residues retained on soil surface;
■ T3 = CA direct maize: no-tillage system with planting done by direct seeding with a dibble/pointed stick, continuous maize monocrop, crop residues retained on soil surface;
■ T4 = CA cowpea rotation: no-tillage system with planting done by direct seeding with a dibble/pointed stick, cowpea-maize-cowpea annual rotation, crop residues retained on soil surface;
■ T5 = CA maize rotation: no-tillage system with planting done by direct seeding with a dibble/pointed stick, maize-cowpea-maize annual rotation, crop residues retained on soil surface;
■ T6 = CA pigeon pea intercropping: no-tillage system with planting done by direct seeding with a dibble/pointed stick, maize-pigeon pea intercrop, crop residues retained on soil surface;
■ T7 = CA cowpea intercropping: no-tillage system with planting done by direct seeding with a dibble/pointed stick, maize-cowpea intercrop, crop residues retained on soil surface;
■ T8 = CA velvet bean intercropping: no-tillage system with planting done by direct seeding with a dibble/pointed stick, maize-velvet bean intercrop, crop residues retained on soil surface.

Weed control consisted of pre-emergent herbicide treatment with glyphosate at 2.5 L/ha followed by weeding with a hand hoe or by handpicking up to three times per season, depending on the level of weed infestation. The crops received 793 and 845 mm of rainfall during the 2018–19 and the 2019–20 growing seasons, respectively (Supplementary Material 1). Fertiliser application, weeding, and insect control were similar as above. More detailed description of this long-term CA trial is provided by Steward et al. (32).

At maturity, out of the four replicate plots of each treatment, three plots of each variety or each CA treatment were randomly selected for grain sampling. All cobs of each net plot were harvested and shelled. Grains were winnowed and weighed to determine yield. Grain yield (in kg/ha) was determined by weighing the shelled dry grains of each plot and dividing that value by the area of the plot. The grains were thoroughly mixed before sampling approx. 0.1 kg of grains into ziplock polyethylene sampling bags. Once in the laboratory, the sample was mixed again, and a representative sample of approx. 500 g was powdered in a grinder (Kitchen Perfected; Lloytron PLC, United Kingdom) to obtain maize flour of 300–500 μm particle size. Maize flour (100–150 g) was sampled in fresh polyethylene bags and shipped for laboratory analysis of minerals.

### Mineral Analysis

The sample preparation protocol was adapted from Phan-Thien et al. (37). Maize flour (600 mg) was weighed in a glass test tube, and 3 ml of 69% HNO_3_ (Hiperpur; Panreac, Spain) and 2 ml of deionized water (Milli-Q; Merck, Spain) were added. The mixture was digested by microwave treatment (Milestone; Ultrawave, Italy) at 240°C and 40 bars for 40 min at 1,500 W. Once digested, it was brought to a final volume of 50 ml with Milli-Q water. Carbon and N were analysed by inductively coupled plasma optical emission spectroscopy (ICP-OES), and the other minerals (F, K, Mg, Mn, P, Se, and Zn) were analysed by inductively coupled plasma mass spectroscopy (ICP-MS) using the analysis parameters from Otero-Romaní et al. (38). Analysis was performed on a PerkinElmer Optima 4600 DV ICP analyser (Waltham, United States). The running parameters were set as follow: plasma flow 15 L/min, auxiliary flow 0.2 L/min, nebulizer flow 0.8 L/min, power 1,300 W, reading distance 15 mm, reading position radial (K) and axial (Mg, Mn, Zn, Fe, and P), integration time 5–10 s, and number of replicates 3. For quantification, standards (Panreac Química SLU, Spain) were prepared in HNO_3_-H_2_O in the same proportion as the samples (matrix-matched calibration standards). Standard reference material GBW10011 was used for recovery and limit determination. Detection and quantification validation parameters, including isotopes used for detection, concentration range of the standards, linearities, recovery percentages, relative standard deviation (RSD) limits of detection (LOD), and limits of quantification (LOQ) are summarised in Table 2 below. Each flour sample was analysed in triplicate.

**Table 2:** Detection and quantification parameters of maize minerals by inductively coupled plasma mass spectroscopy (ICP-MS).

**Mineral**	**K**	**Mg**	**Mn**	**Zn**	**Fe**	**P**	**Se**
Isotope	39	24	55	66	56	31	78
Standard concentration range	0.5–50	0.5–50	1–100	10–1,000	10–1,000	1–50	0.2–10
Standard concentration unit	mg/L	mg/L	μg/L	μg/L	μg/L	mg/L	μg/L
Linearity	0.9999	0.9999	1.0000	0.9998	1.0000	0.9995	0.9998
Recovery (%)	93.7	99.8	74.0	96.8	65.9	68.7	95.4
Relative standard deviation (RSD) (%)	6.4	6.5	5.9	7.6	6.1	6.2	14.2
Limit of detection (LOD) (mg/kg)	0.8111	0.0262	0.00276	0.0514	0.00178	2.604	0.0392
Limit of quantification (LOQ) (mg/kg)	36.198	0.173	0.00779	0.3098	0.2448	17.11	0.2384

Nutrient content was corrected for moisture content, and determined with the Association of Analytical Communities (AOAC) Method 991.39 (39). C and N content was expressed in g/100 g dry weight, while that of Fe, K, Mg, Mn, P, Se, and Zn was in mg/100 g dry weight. The nutrient yield was calculated by multiplying nutrient content with corresponding grain yield, and expressed in mass (g or kg) nutrient per area (ha).

### Contribution to the Dietary Requirements of Women and Children

Maize consumption for adults (337 g per capita per day) was taken from FAO data (1), and consumption data for children under 5 years (136 g per capita per day) was obtained from AFRICAP Household Vulnerability Survey in Balaka and Nkhotakota districts of Malawi in 2019 and 2021 (unpublished data). The protein content of maize was obtained by multiplying nitrogen content by 6.25 (40) and expressed in g/100 g dw. The product of grain mineral concentration and daily flour intake provided an estimate of the daily intake of each nutrient. Daily intakes were compared with the dietary reference intakes (DRIs) for children (1–3 years) and women (19–50 years) obtained from the Institute of Medicine (41) to estimate the percentage of dietary requirement of Malawian women and children in potentially met by the intake of the maize varieties and upon CA treatments (42).

### Statistical Analysis

Mineral analyses were performed in triplicates. Data were statistically analysed using IBM SPSS Statistics v 26. ANOVA and *post-hoc* Tukey's honest significant difference (HSD) test were performed to determine the significance of difference among means of mineral, yield, or DRI contribution of the maize varieties and CA treatments. The difference between the OPVs and hybrids was also assessed by an unpaired two-tailed *t*-test. Year-to-year variation of the studied parameters for each CA treatment was determined by a paired two-tailed *t*-test. Level of significance of 0.05 was considered.

## Results and Discussion

### Grain Yield, Mineral Content, and Nutrient Yield of Maize OPVs and Hybrids

#### Grain Yield

**Figure 1:**
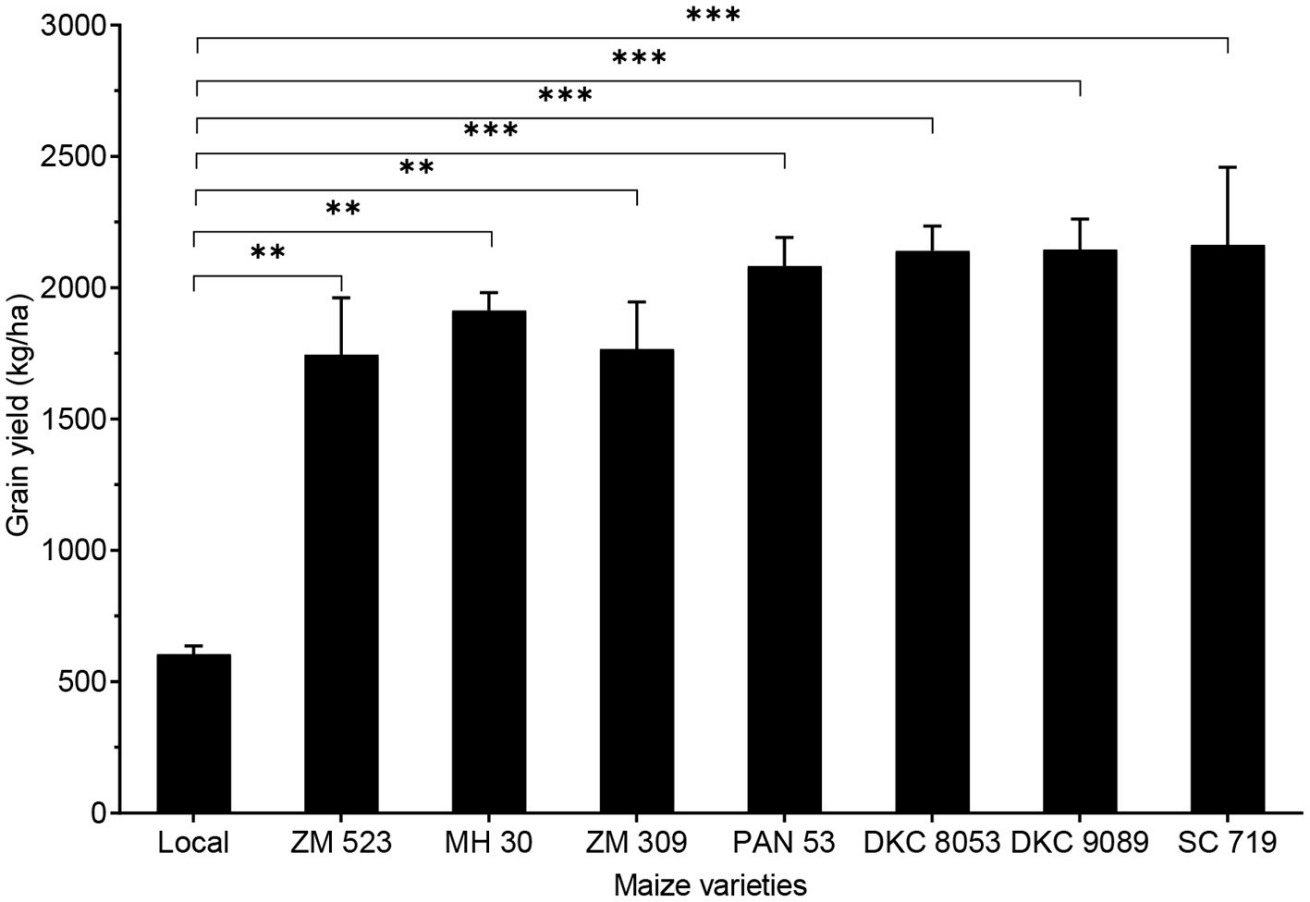
Grain yield of eight maize varieties grown in Malawi. Error bars represent standard error of mean (SEM). ** and *** mean significant at 0.01 and 0.001, respectively, Tukey's honest significant difference (HSD) test.

Grain yield significantly differed among the maize varieties (*p* < 0.001), and the lowest yield (604.6 kg/ha) was obtained from the local variety *Kanjerenjere*, all the others showed yield values 2.9- to 3.6-fold higher, with the highest (2,163.3 kg/ha) obtained with SC 719 (Figure 1). The grain yields of the eight maize varieties obtained in this study are on par with the average national maize yield of Malawi (1,782.4 kg/ha) and the Eastern African yield (1,948.9 kg/ha), as well as the African yield (2,011.5 kg/ha). However, these maize productivities are still very low when compared to the average maize yield in Southern Africa (4,581.8 kg/ha) and worldwide (5,823.8 kg/ha) (1). Maize yield in many SSA countries is low, and reducing yield gap (difference between actual and potential yields) necessitates the use of high-yielding varieties, improved on-field pest control and crop disease prevention, and continued promotion of optimised modern agricultural inputs (43, 44).

#### Mineral Content

**Figure 2:**
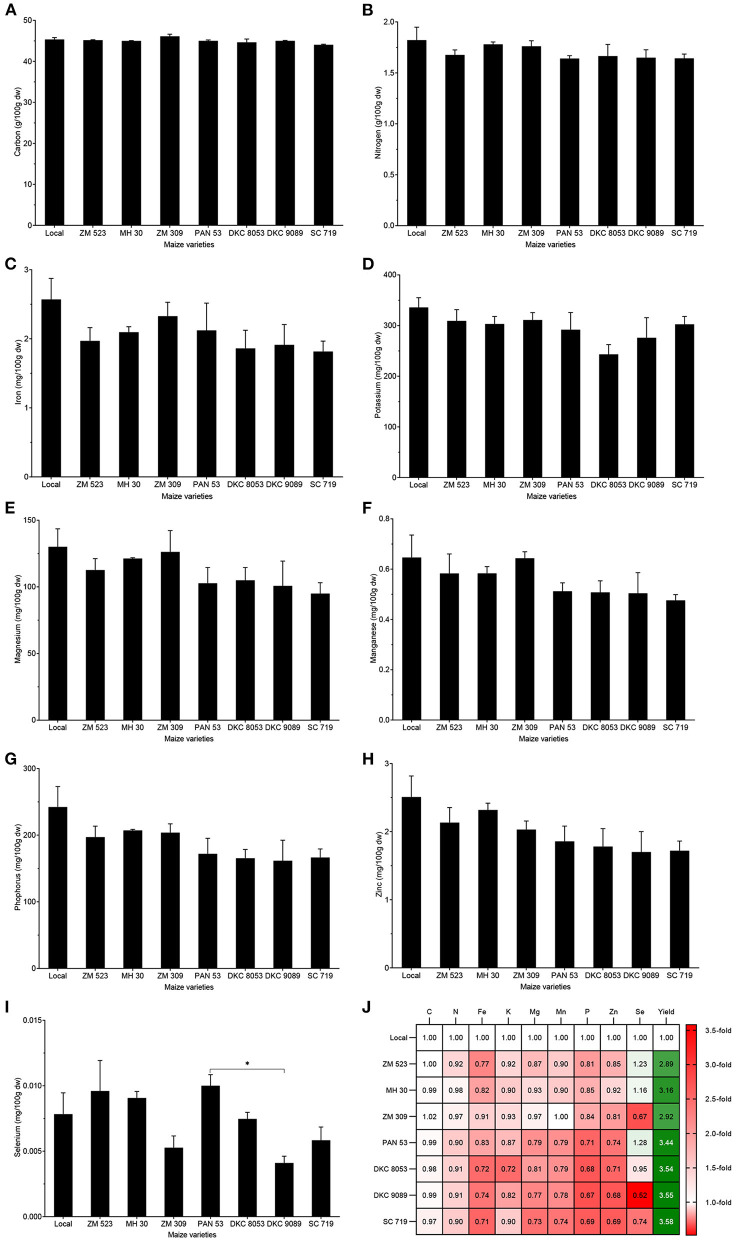
Mineral content of eight maize varieties grown in Malawi. Error bars represent SEM. * means significant at 0.05, Tukey's HSD test. Values in plate **(J)** are the number of fold-change compared to local variety.

Carbon content varied between 44 and 46.1%, and did not significantly differ among the eight maize varieties. The content of N in the grain was between 1.6 and 1.8%, and was not significantly different among the varieties. Grain Fe content (1.8–2.6 mg/100 g), K content (243.3–335.9 mg/100 g), Mg content (95.0–130.1 mg/100 g), Mn content (0.48–0.65 mg/100 g), P content (161.7–242.3 mg/100 g), and Zn content (1.7–2.5 mg/100 g) did not significantly differ among the eight maize varieties. Grain Se content significantly differed among the maize varieties (*p* = 0.024), the lowest content (0.004 mg/100 g) was obtained from DKC 9089, while variety ZM 523 showed the highest Se content (0.01 mg/100 g). Overall, grain Se content significantly differed among the eight varieties, while the content of C, N, Fe, K, Mg, Mn, P, and Zn were not significantly different, although the varieties local and MH 30 seemed to show higher values (Figure 2).

#### Nutrient Yield

**Figure 3:**
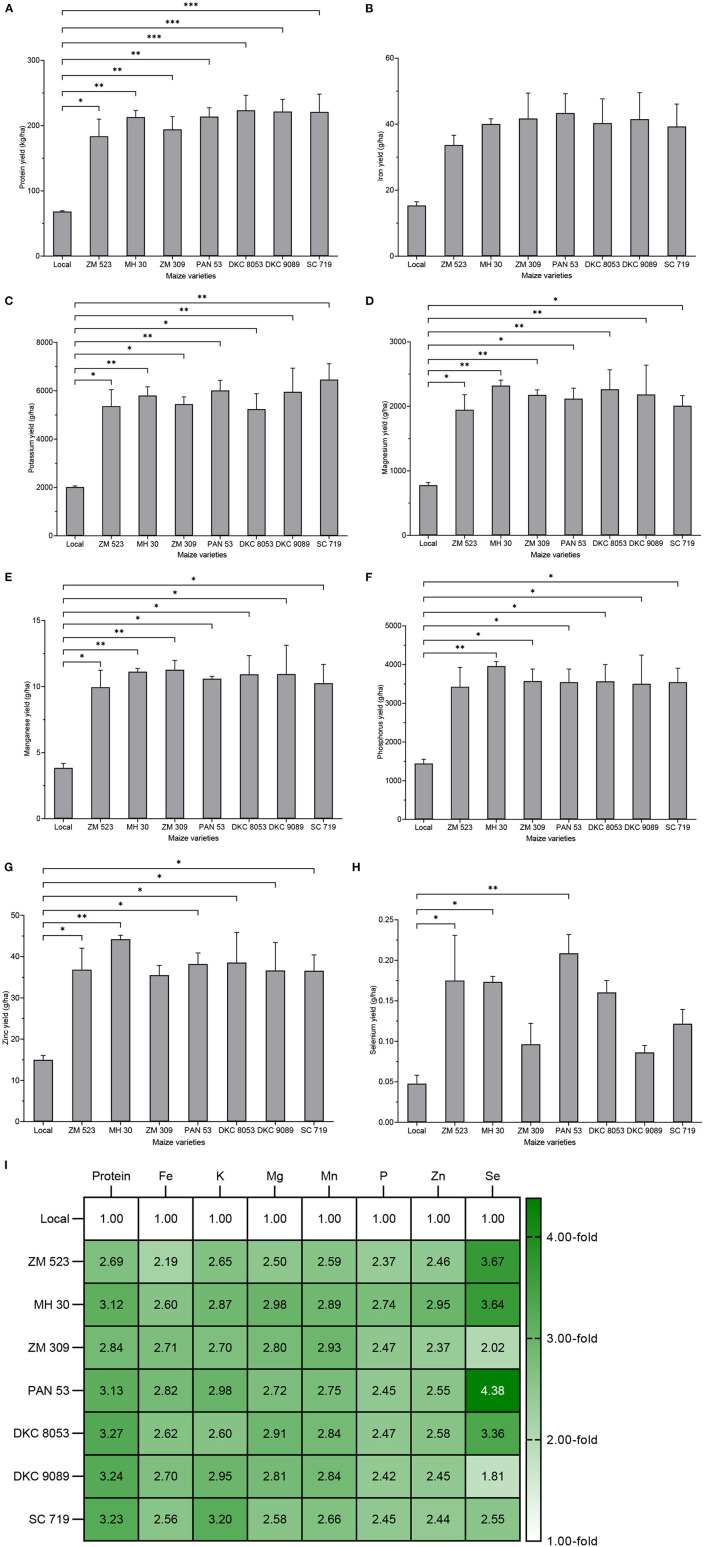
Nutrient yield of eight maize varieties grown in Malawi. Error bars represent SEM. *, **, and *** mean, significant at 0.05, 0.01, and 0.001, respectively, Tukey's HSD test. Values in plate **(F)** are the number of fold-change compared to local variety.

When integrating grain yield, the calculated protein yield of the maize varieties was significantly different (*p* < 0.001): the local variety yielded the lowest (68.3 kg/ha), while the highest yield (223.6 kg/ha) was obtained from DKC 9089. Fe yield varied from 15.4 to 43.4 g/ha, but no significant difference was found. Potassium yield showed significant difference (*p* = 0.002), and varieties local (2,020 g/ha) and MH 30 (6,462.9 g/ha) yielded the lowest and highest K content, respectively. Mg yield was significantly different (*p* = 0.005), the lowest yield (778.3 g/ha) was obtained with the local variety, while the highest yield (2,321.9 g/ha) was recorded for variety MH 30. Mg yield was significantly different (*p* = 0.007), the lowest and the highest yield (3.9 and 11.3 g/ha) were obtained with varieties local and ZM 309, respectively. There was a significant difference in the P yield of the eight maize varieties (*p* = 0.017), the lowest and the highest values were recorded in varieties local (1,145.3 g/ha) and MH 30 (3,960.7 g/ha), respectively. Zn yield was significantly different (*p* = 0.013), the lowest and the highest yield, 15 and 44.2 g/ha, were obtained with local and MH 30 varieties, respectively. Selenium yield was significantly lower (*p* =0.005) for the local variety (0.05 g/ha), while variety PAN 53 showed the highest yield (0.21 g/ha). To sum up, as the grain yield of the local variety was 2.9- to 3.6-fold lower, the resulting nutrient yields were significantly lower in the local variety (except for Fe), and varieties MH 30, PAN 53, and SC719 provided the best yield for most of the nutrients (Figure 3).

Parallel to the grain yield, the nutrient content of grain is another important quality parameter. Compared to the composition of unfortified raw whole white maize flour listed in the Malawi food composition table (food item Reference MW01_0019) (45), the maize varieties in this study showed similar contents of N, K, and P, lower contents of Fe, and higher amounts of Mg and Zn. Our results of content of Fe, Mg, Zn, and Se also agree with a previous report (22) on mineral concentration in maize grain from calcareous and non-calcareous soils across Malawi. However, we report Se concentrations much lower than values recorded on Se-biofortified maize, but higher than when Se was not supplied to the soil in a similar agroecological setting in Malawi (46).

Many factors can influence the composition of maize kernels, such as genetic background of each variety, environmental growth conditions, plant age, geographic location, soil type and nutrients, and agronomic practise (23, 47, 48). The genetic background of maize varieties and cultivars significantly affect the remobilization efficiency of the nutrients in different parts of the plant, and the accumulation of these nutrients in the grain (49). Nutrient concentration in cereal grains depends mostly on soil and environmental parameters, such as soil pH, soil organic matter, temperature, rainfall, and topography (50). In Malawi, it was shown that the maize grain content of Ca, Cu, Fe, Mg, Se, and Zn were higher from plants grown on calcareous soils than those on low-pH soils (such as *Alfisol* of the Chitedze experimental site), which are more widespread in the country (22). Differences in root architecture between varieties and their level of root colonisation by symbiotic arbuscular mycorrhizal fungi may also justify the difference in their nutrient composition. In fact, mycorrhiza-associated maize plants showed higher grain yield (51) and enhanced proximate composition (crude protein, fat, moisture, and ash) of maize grain (52), but the importance of symbiotic fungi in the African context remains poorly understood. The reported information on maize grain composition of different varieties grown in Malawi in this study is an important resource to define nutrient yield baseline data in the Malawian context and encourage plant breeding efforts for nutritionally superior maize varieties. Considering that Fe, Zn, and Se are among the MNDs of most concern worldwide, varieties PAN 53 and MH 30 could be good high-yielding candidates for these minerals and should be tested in other sites. Besides, the tested maize varieties may provide a better reflection of the composition of maize consumed and improve the estimates of mineral deficiency risks in Malawi (14).

### Effect of Conservation Agriculture on Grain Yield, Mineral Content, and Nutrient Yield

#### Grain Yield

**Figure 4:**
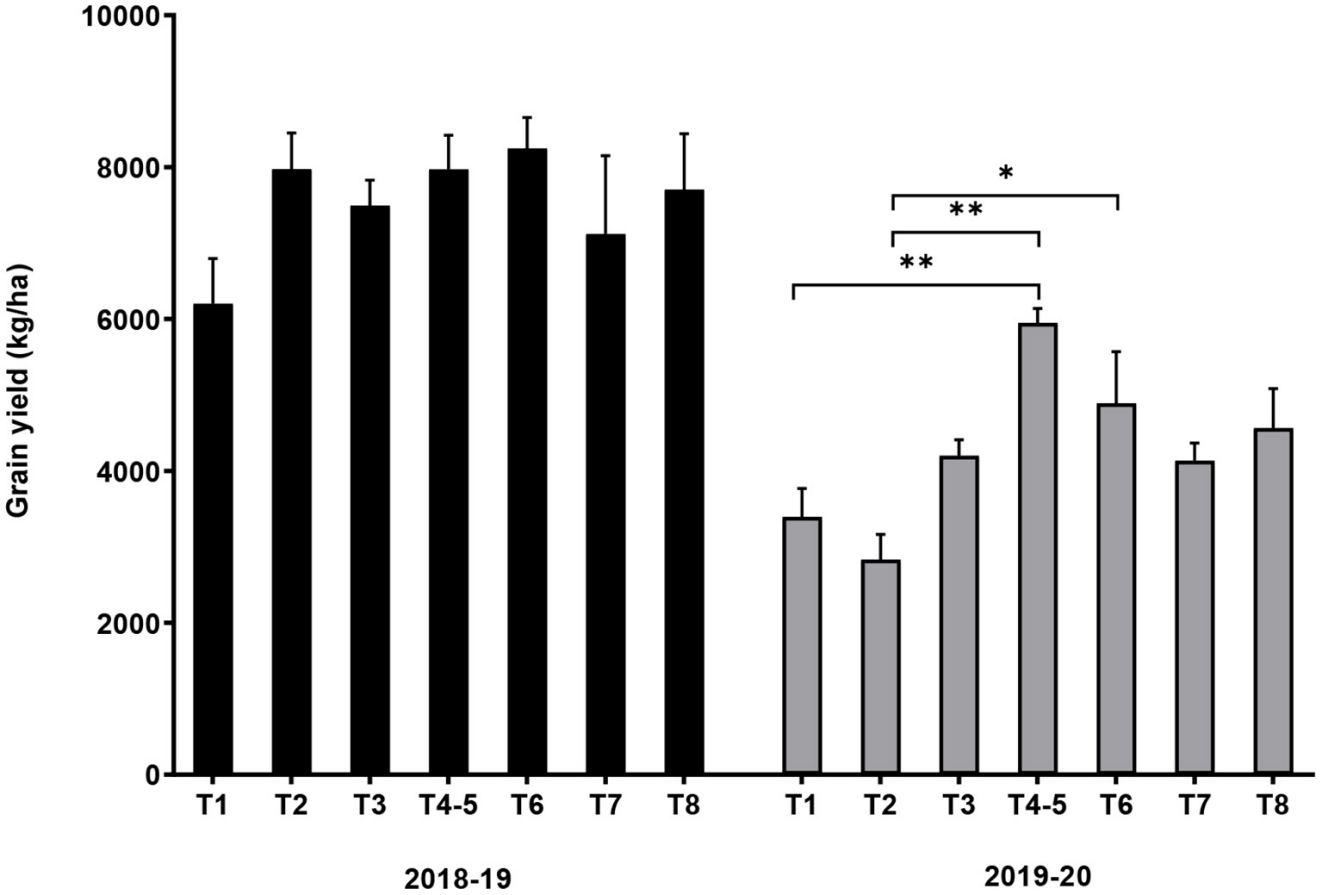
Effect of conservation agriculture on grain yield of maize variety DKC 9089 during two seasons of field trials. Error bars represent SEM. *and **mean significant at 0.05 and 0.01, respectively, Tukey's HSD test. Treatments: T1 = conventional agriculture; sole maize. T2 = conservation agriculture (CA); basin sowing; sole maize. T3 = CA; dibble stick sowing; sole maize. T4–5 = CA; dibble stick sowing; maize-cowpea rotation. T6 = CA; dibble stick sowing; maize-pigeon pea intercrop. T7 = CA; dibble stick sowing; maize-cowpea intercrop. T8 = CA; dibble stick sowing; maize-velvet bean intercrop.

Conservation agriculture treatments with variety DKC 9089 increased grain yield, with a significant augmentation (*p* = 0.002) of 1.2- to 1.8-fold obtained during the 2019–20 trial, except for treatment T2. The CA treatment T4–5 involving dibble stick sowing and maize-cowpea rotation resulted in almost twice the yield of the T1 (conventional agriculture) control (Figure 4).

#### Mineral Content

**Figure 5:**
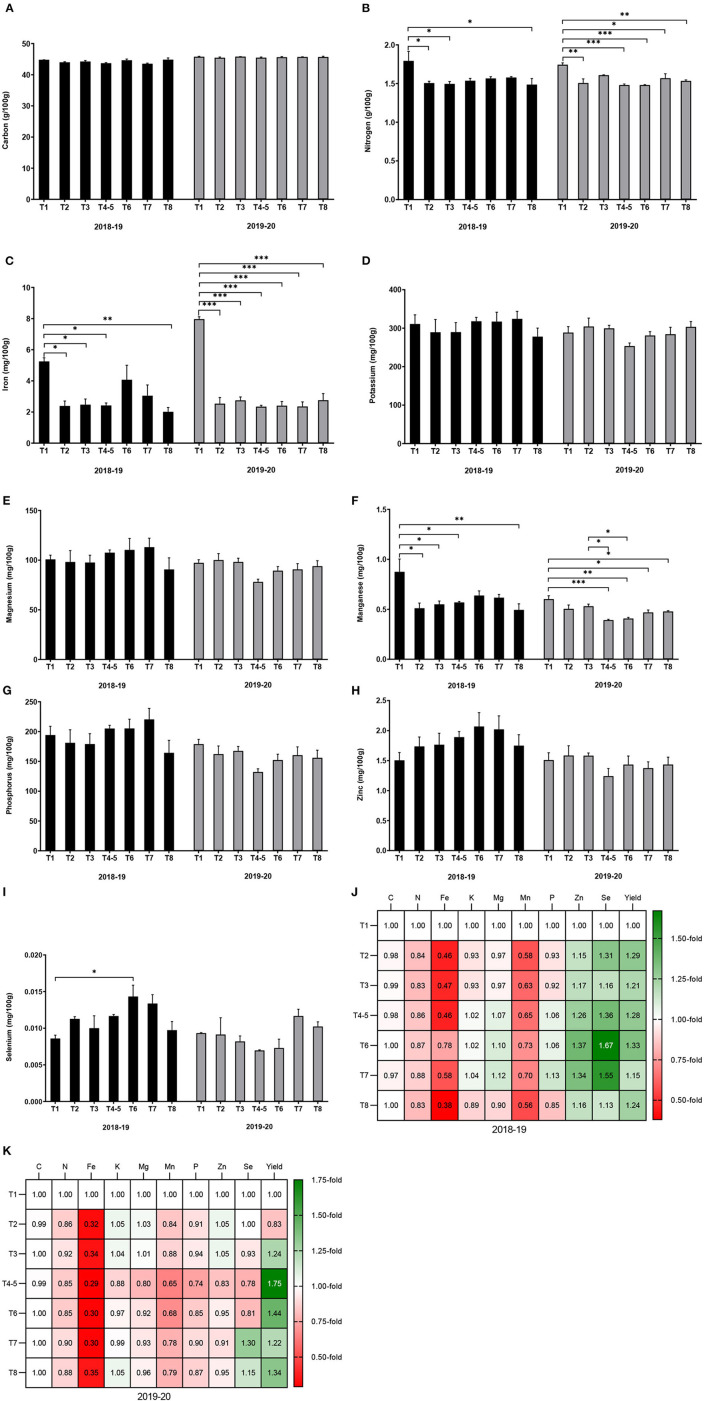
Effect of conservation agriculture on the nutrient content of maize variety DKC 9089 during two seasons of field trials. Error bars represent SEM. *, **, and *** mean, significant at 0.05, 0.01, and 0.001, respectively, Tukey's HSD test. Values in **(J,K)** are the number of fold-change compared to treatment T1 (conventional practise). Treatments: T1 = conventional agriculture; sole maize. T2 = conservation agriculture (CA); basin sowing; sole maize. T3 = CA; dibble stick sowing; sole maize. T4–5 = CA; dibble stick sowing; maize-cowpea rotation. T6 = CA; dibble stick sowing; maize-pigeon pea intercrop. T7 = CA; dibble stick sowing; maize-cowpea intercrop. T8 = CA; dibble stick sowing; maize-velvet bean intercrop.

There was also no change in grain C content as a result of CA treatments for both cropping seasons tested. Under CA treatments, a significantly slight decrease (1.1- to 1.2-fold) of N content was observed for the two seasons tested (*p* = 0.033 for 2018–19 and *p* < 0.001 for 2019–20). Conservation agriculture treatments led to a significant decrease in grain Fe content by 1.3- to 2.8-fold for the 2018–19 trial (*p* = 0.004) and by 2.9- to 3.4-fold for the 2019–20 trial (*p* < 0.001). Treatments T6 and T7 (maize-legumes intercrop) did not significantly differed from the T1 control in the 2018–2019 season. During the two-season trials, there was no noticeable change in the K, Mg, and P contents of maize grains between the CA treatments. Grain Mn content was reduced by all the CA treatments during the two tested farming seasons: a 1.2- to 1.8-fold decrease was observed in 2018–19 (*p* = 0.012), and the decrease was between 1.1- and 1.5-fold in 2019–20 (*p* = 0.001). The Zn content in the maize grain was not significantly affected by the CA treatments, despite the noticeable increase in the 2018–19 trial. All the CA treatments significantly increased Se content in the maize grains by 1.1- to 1.7-fold in the 2018–19 trial (*p* = 0.027); treatment T6 (dibble stick sowing and maize-pigeon pea intercrop) showed the highest increase. In the 2019–20 trial, no significant change was observed (Figure 5).

#### Nutrient Yield

**Figure 6:**
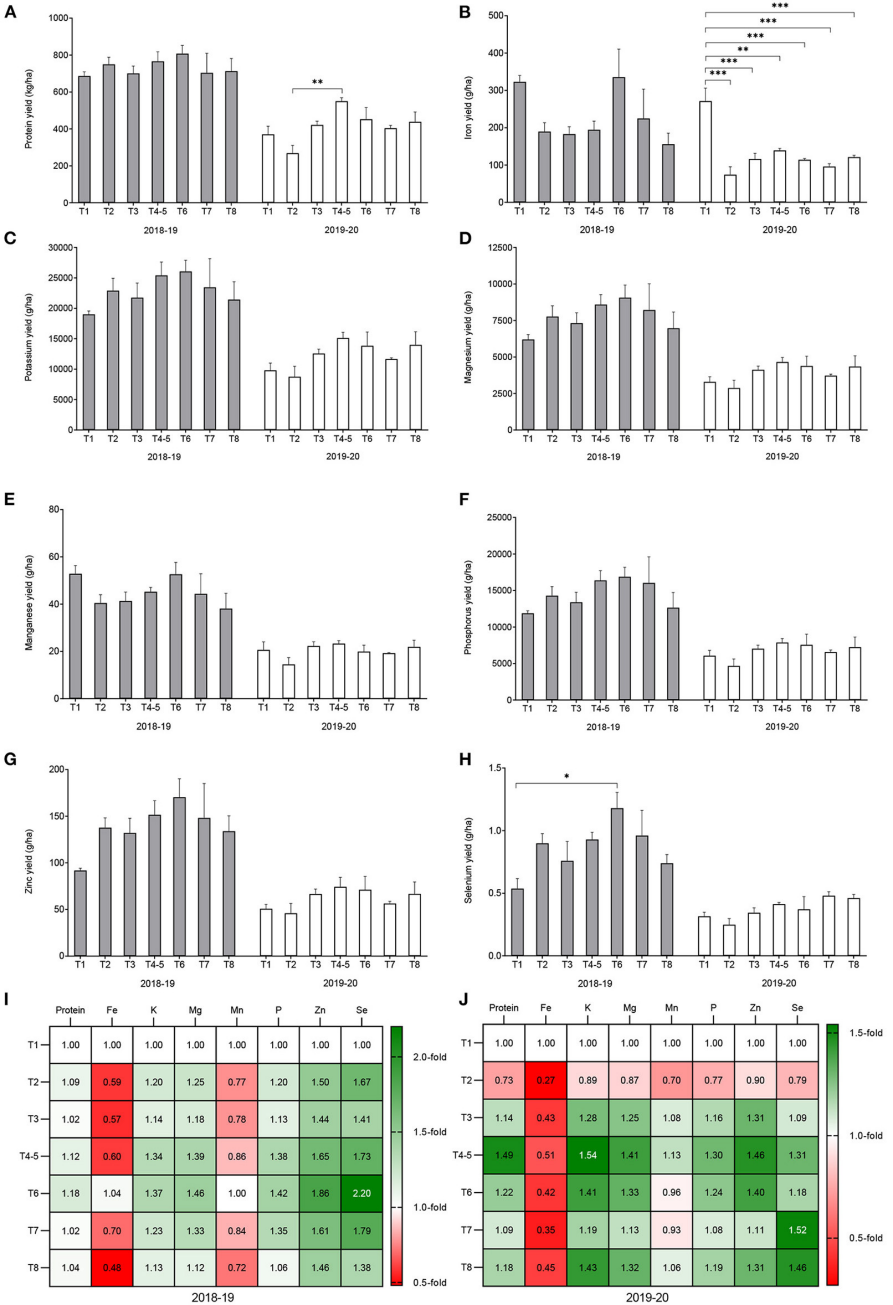
Effect of conservation agriculture on nutrient yield of maize variety DKC 9089 during two seasons of field trials. Error bars represent SEM. *, **, and *** mean, significant at 0.05, 0.01, and 0.001, respectively, Tukey's HSD test. Values in Figures 5J,K are the number of fold-change compared to treatment T1 (conventional practise). Treatments: T1 = conventional agriculture; sole maize. T2 = conservation agriculture (CA); basin sowing; sole maize. T3 = CA; dibble stick sowing; sole maize. T4–5 = CA; dibble stick sowing; maize-cowpea rotation. T6 = CA; dibble stick sowing; maize-pigeon pea intercrop. T7 = CA; dibble stick sowing; maize-cowpea intercrop. T8 = CA; dibble stick sowing; maize-velvet bean intercrop.

As a result of yield increase, the CA treatments resulted in increase in protein yield, which was significant in the 2019–20 trial (*p* = 0.01), except for treatment T2. Conversely, significant decreases in Fe yield affecting all the CA treatments were recorded with the 2019–20 trial (*p* < 0.001). With the exception of T2 in 2019–20, all the CA treatments increased K yield, but gains were not statistically significant. The yield of Mg increased with all the CA treatments but not significantly. There was no significant change in Mn yield for all the treatments during the two season trials. Although P and Zn yield increased under the CA treatments during the two seasons of trials, the difference with the control was not significant. As observed with Se content, Se yield significantly increased in the first trial season (*p* = 0.034) (especially for T6) (Figure 6).

Overall, the CA treatments resulted in a significant increase in grain Se content but did not affect C, K, Mg, P, and Zn, and decreased N, Fe, and Mn in the grain. The CA treatments lead to an increased grain yield, resulting in a significantly higher yield of proteins and Se, had no effect on the yield of K, Mg, Mn, P, and Zn, and reduced yield of Fe. Our results on grain yield agree with many previous reports that showed that CA maize yields outperformed conventional practise (53–58). In this study, a higher yield increase was obtained, with 14.8–33% in 2018–29 and 21.7–75.2% in 2019–20 (excludingT2). Other studies demonstrated that sole maize out-yields all intercropping (59), and that in CA systems, intercropping leads to 5% reduction in maize yield compared to no-till monocropping because of competition between maize and intercropped grain legumes (35). This result is in disagreement with our study: we recorded no significant difference between yields of the no-till CA monocropping treatments (T2 and T3) and the intercropped treatments (T7 and T8); and in the case of maize-pigeon pea intercrop treatment T6, the yield was 1.7-fold higher than T2. Likewise, we observed enhanced protein yields under CA treatment involving crop rotation, while (35) obtained higher protein yield when maize was intercropped with a grain legume. A previous study (46) has shown that CA treatments have little effect on Se uptake by maize, while our work shows 1.1- to 1.7-fold higher Se levels under conventional practise in the 2018–19 trial. These observations suggest that maize yield response to CA depends on the combination of CA practises implemented, duration of CA implementation, location, genetic variability, and year-to-year changes in climatic conditions.

The mechanism by which CA influences maize yield and some nutrients in the grain has not been established yet. It can be explained by different factors, for instance, by enhancement of maize plant resistance to drought stress, especially at anthesis, a growth stage when the plant is most climate-sensitive (32). Furthermore, long-term maize-based CA systems also modify soil hydraulic properties by increasing total porosity, fine pores for water storage, and plant available water capacity (PAWC), resulting in improved soil structure for plant growth (33). The status and management of soil nutrients determine both crop productivity and nutrient concentration in plant parts consumed as food and feed. In fact, the two key factors affecting grain yield and nutrient concentration in maize grain are the availability of soil nutrients for the plant and plant genotype (60, 61). Consequently, soil nutrient status has significant implications on human health (62). Lack of adequate minerals in the soil can lead to food crops deficiency in nutrients and result in deficiency of such nutrients in humans (63). Therefore, there is a need to understand the impact of CA on soil minerals and its effect on the content of minerals in plants and grains.

### Effect of Seasons by Conservation Agricultural Practises on Maize Grain Yield, Mineral, and Nutrient Yield

The differences in maize grain yield and mineral content observed between the 2018–19 and 2019–20 seasons are statistically assessed, and the results are summarised in Table 3. For the majority of the CA treatments, the grain yields were significantly higher in 2018–19 than in 2019–20. Carbon content just slightly differed between the two trials, but the difference was significant for almost all the treatments. Nitrogen content was stable during the two seasons. The iron content of control treatment T1 was much higher in 2019–20 than in 2018–19; all the other treatments did not show any significant change during the two seasons. For K, Mg, Zn, and Se, only the contents of treatment T4–5 (CA; dibble stick sowing; maize-cowpea rotation) were higher in the first season, and the values remained stable for all the other treatments. The contents of Mn and P were also stable for the majority of the treatments, except for T4–5 and T6 (CA; dibble stick sowing; maize-pigeon pea intercrop) where higher contents were recorded in the first season.

**Table 3:** *P*-values and significance of year-to-year variation in maize grain yield and mineral content between the 2018–19 and 2019–20 conservation agriculture trials.

**Treatment**	**Yield**	**Carbon**	**Nitrogen**	**Iron**	**Potassium**	**Magnesium**	**Manganese**	**Phosphorus**	**Zinc**	**Selenium**
T1	0.008^**^	0.018^*^	0.766	0.020^*^	0.384	0.353	0.202	0.339	0.948	0.284
T2	0.019^*^	0.024^*^	0.997	0.453	0.328	0.772	0.764	0.176	0.020^*^	0.496
T3	0.011^*^	0.042^*^	0.085	0.626	0.744	0.954	0.521	0.653	0.349	0.325
T4-5	0.035^*^	0.050	0.234	0.775	0.020^*^	0.023^*^	0.007^**^	0.004^**^	0.020^*^	0.002^**^
T6	0.065	0.025^*^	0.082	0.136	0.198	0.203	0.033^*^	0.022^*^	0.051	0.123
T7	0.095	0.010^*^	0.900	0.418	0.216	0.101	0.057	0.067	0.075	0.405
T8	0.039^*^	0.391	0.587	0.401	0.108	0.717	0.802	0.508	0.106	0.590

* and ** *mean significant at 0.05 and 0.01, respectively, paired two-tailed t-test. Treatments: T1 = conventional agriculture; sole maize. T2 = conservation agriculture (CA); basin sowing; sole maize. T3 = CA; dibble stick sowing; sole maize. T4–5 = CA; dibble stick sowing; maize-cowpea rotation. T6 = CA; dibble stick sowing; maize-pigeon pea intercrop. T7 = CA; dibble stick sowing; maize-cowpea intercrop. T8 = CA; dibble stick sowing; maize-velvet bean intercrop*.

Given that maize plant is sensitive to drought stress, variation in climatic conditions, especially rainfall, during the two trial seasons may explain the observed difference. Similar disparities in different years of agronomic trials have been reported in different research stations of Malawi (64). Previous studies have also shown that rainfall and drought during cropping season can influence maize physiological response and agronomic performance under CA conditions (32, 35, 53). A longitudinal analysis of the long-term CA experiment in Malawi showed a season effect on yield, and highlighted the strong interaction between CA treatments and climatic conditions (53). Therefore, stability of the effect of CA across years should be considered both for agronomic performance and grain nutrient parameters.

### Comparison of Open-Pollinated Varieties and Hybrid Maize

**Figure 7:**
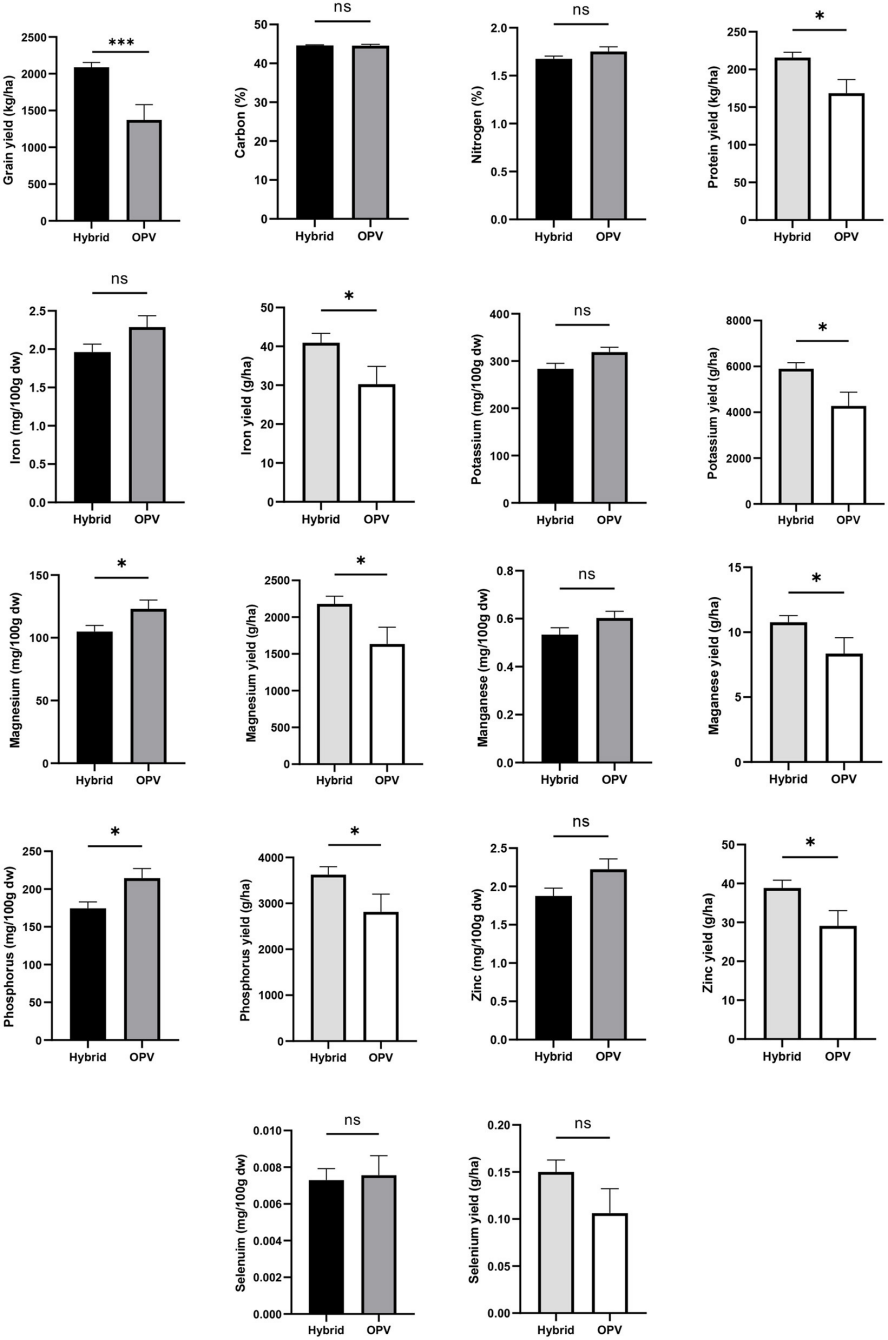
Comparison of open-pollinated varieties and hybrid maize grown in Malawi for their grain yield, mineral content, and nutrient yield. Error bars represent SEM. ns, * and ***mean significant at 0.05 and 0.001, respectively, two-tailed unpaired *t*-test.

The OPVs showed higher grain mineral content than the F1 hybrids, with significant differences for Mg (*p* = 0.042) and P (*p* = 0.013). However, the average grain yield of the hybrids was 1.5-fold significantly higher (*p* < 0.001) than that of the OPVs. Consequently, the hybrids significantly outperformed the OPVs in all the nutrient yields, except for Se in which the difference was not significant (Figure 7).

Our results on yield are similar to those reported by Pixley and Bänziger (65), which showed that under typical growing conditions of eastern and southern Africa, elite hybrids produced 18% more grain than elite POVs in the first generation, but that in the second generation (recycled seeds), OPVs yielded 32% more than hybrids. The results on mineral composition corroborate another finding (26), which demonstrated that the chemical composition, bioactive compounds, and physical properties (technological quality) of OPV maize were superior than those of hybrids.

These observations can be an indication that these F1 hybrid varieties grown in Malawi were developed essentially for high yield, and that this is detrimental to mineral content and nutritional value. Our results suggest that if looking for varieties for breeding of nutrient-rich maize, the OPVs appear as good candidates. Reproduction of OPVs is by cross-pollination either between two plants (with the help of wind or insects) or from separate flowers on the same plant. Taking advantage of the many recent advances in breeding techniques, nutritionally improved OPVs of maize that also possess several desirable alleles, such as resistance to biotic and abiotic stress factors, are being developed. The OPVs also have the advantage of being cheaper than FI hybrid varieties, and, unlike them, their seeds can be replanted for about 3–4 years without considerable yield loss, which ultimately increases the accessibility to improved seeds by resource-poor smallholder farmers in SSA (66).

### Contribution to the Dietary Requirements of Malawian Children and Women

**Figure 8:**
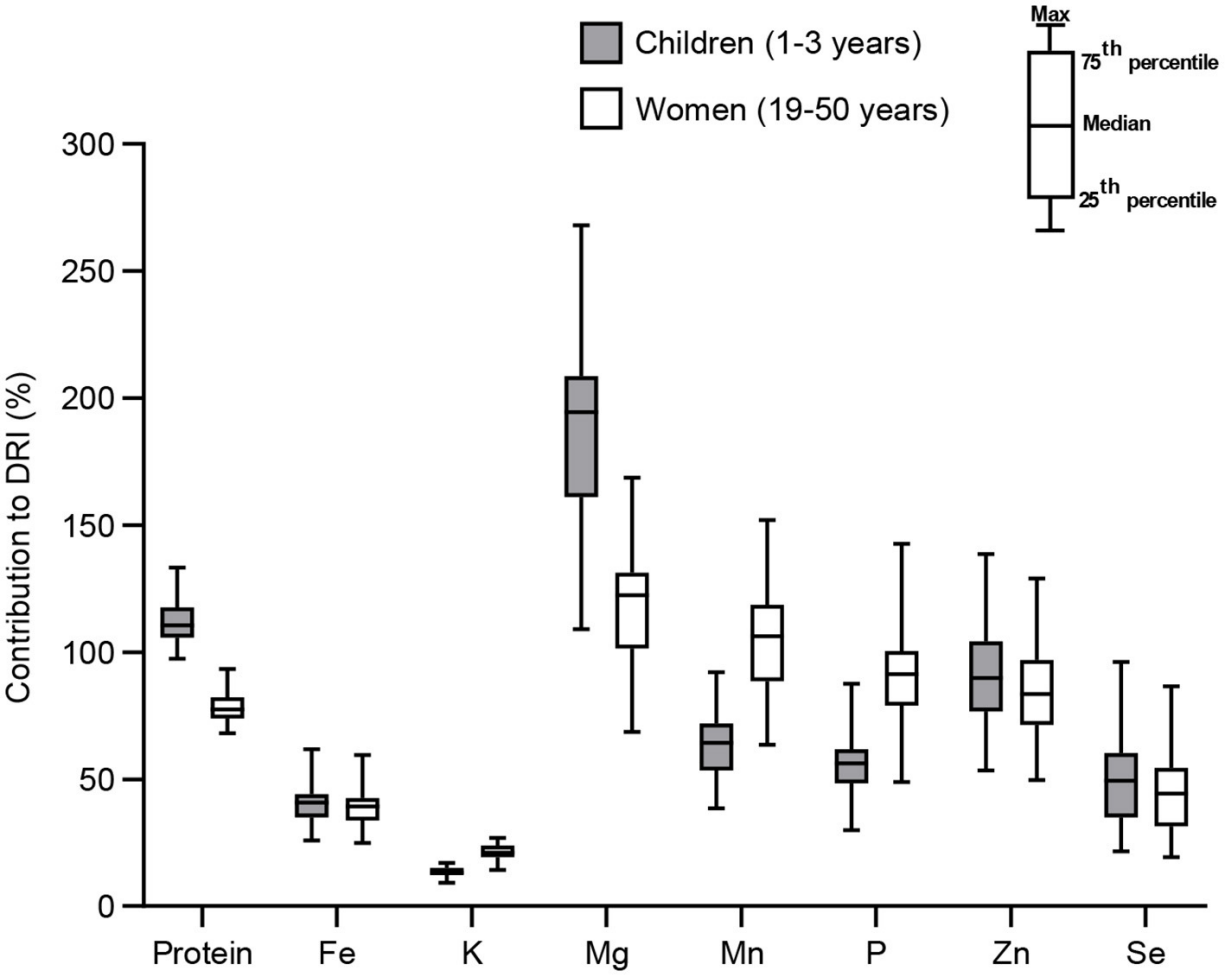
Contribution of maize varieties grown in Malawi to the nutrient dietary requirements of Malawian children and women.

The overall contribution to DRIs of each mineral of the eight maize varieties tested in this study is presented (Figure 8). On average (median value), the maize whole flour from the grain of these varieties could contribute to 110.5 and 77.4% of protein DRIs of Malawian children and women, respectively, 40.8 and 39.3% of Fe needs, 13.3 and 21% for K, 194.5 and 122.4% for Mg, 64.3 and 106.3% for Mn, 56.1 and 91.4% for P, 89.8 and 83.4% for Zn, and finally 49.3 and 44.4% for Se. Except for Se in which the contribution of variety ZM 253 was significantly higher than that of variety ZM 309 (*p* = 0.048), no other significant difference was found among the maize varieties in their contribution to the DRIs for Malawian children and women.

These estimates of contribution to nutrient needs using local maize composition data that can capture environmental influences provide better information on nutrient intakes and estimation of deficiency risks among populations than those habitually produced using food supply data (22, 50). A recent report shows that most households in Malawi could receive <25% of Se, 25–50% of Fe, and <50–75% of their Zn requirements from a typical consumption pattern (50). This supports our above results for Fe but disagrees with our values for Se and Zn, probably because of the effect of different geographical locations in both studies (14, 50). Our results on high supply of Mg by the eight Malawian maize varieties (194.5 and 122.4% of children and women DRIs, respectively) are aligned with previous results obtained in Malawi (22), where Mg supply from maize grown in different soils in the country represented 355–389% of adult female needs. These suggest a low risk of dietary Mg deficiency in Malawi previously evidenced, based on national food supply data (63, 67).

It was reported that maize foods consumed in eastern and southern African countries could satisfy <1/4 of women requirements for many nutrients; half of the children demand of Mg, between 1/4 and 1/2 of their needs in protein and Zn, but just a minor contribution in their daily requirement for Ca, Na, and Se (4). Although our above results tend to indicate that Malawian maize is more nutritious than previously reported, it should be also considered that the consumption value of maize in Malawi is one of the highest in Africa and worldwide. We have only assumed the mineral content of raw whole maize flour, while it is known that processing operations common in SSA (like dehulling, degerming, milling, refining, and polishing) and cooking alter the nutritional composition of maize (24, 68). These processing operations could also influence the nutrient availability from maize when consumed (68). Likewise, a high prevalence of malnutrition indicators linked to MNDs was found in the country: stunting, underweight, and wasting affected 37.1, 12.8, and 2.7% of children under 5 years, respectively (19, 20); and 28% of preschool children, 21% of school-aged children, and 21% of non-pregnant women of reproductive age suffered from anaemia, which is linked to Fe deficiency (15).

This study showed that maize varieties grown in Malawi could only contribute approximately 40% of Fe DRI, and 44–49% for Se. On the other hand, risk of and actual Fe, Ca, Zn, and Se deficiency in women and children are health concern in Malawi (14–16). This suggests that these population needs to complement their Fe and Se needs from other sources by diversifying the diet, or that maize could be fortified or biofortified with these minerals. Besides, although this study found that the staple maize in Malawi could contribute 89.8 and 83.4% of Zn requirement of children and women, Zn deficiency still affects 60% of children and 63% of women in the country (15). The paradoxical high occurrence of Zn deficiency and considerable Zn intake from maize can be due to loss of nutrients during processing and cooking, low food diversification, and reduced nutrient bioavailability; a similar observation was reported in other eastern and southern Africa countries (4). Besides, the low K intake from maize by adult women observed in our study (only 1/5 of K needs met) could hinder the benefits of K for health, such as blood pressure reduction in adults (low risk of stroke and coronary heart disease), protection against age-related bone loss, and reduction of kidney stones (69).

These observations are particularly important in the current pandemic crisis. A recent review shows that together with vitamins A, Bs, C, D, and E, minerals Zn, Se, I, Cu, and Fe play a role in the mobilisation of immune responses to viral infections, such as SARS-CoV-2, which is responsible for the current coronavirus disease 2019 (COVID-19). In infected persons, MNDs contribute to the emergence of more virulent strains and, with dysfunction of the immune response, may contribute to the morbidity of COVID-19 infection (70). Additionally, vitamin D deficiency worsens the clinical outcome of patients with COVID-19 (71), while Mg could help in absorption, synthesis, and function of vitamin D in the body, and a Mg deficiency negatively affects vitamin D status (72–75). This implies that the high Mg supply from Malawian maize could contribute in fighting against the burden of COVID-19 disease in the population.

### Effect of Conservation Agriculture on Maize Food and Nutrition Security

**Figure 9:**
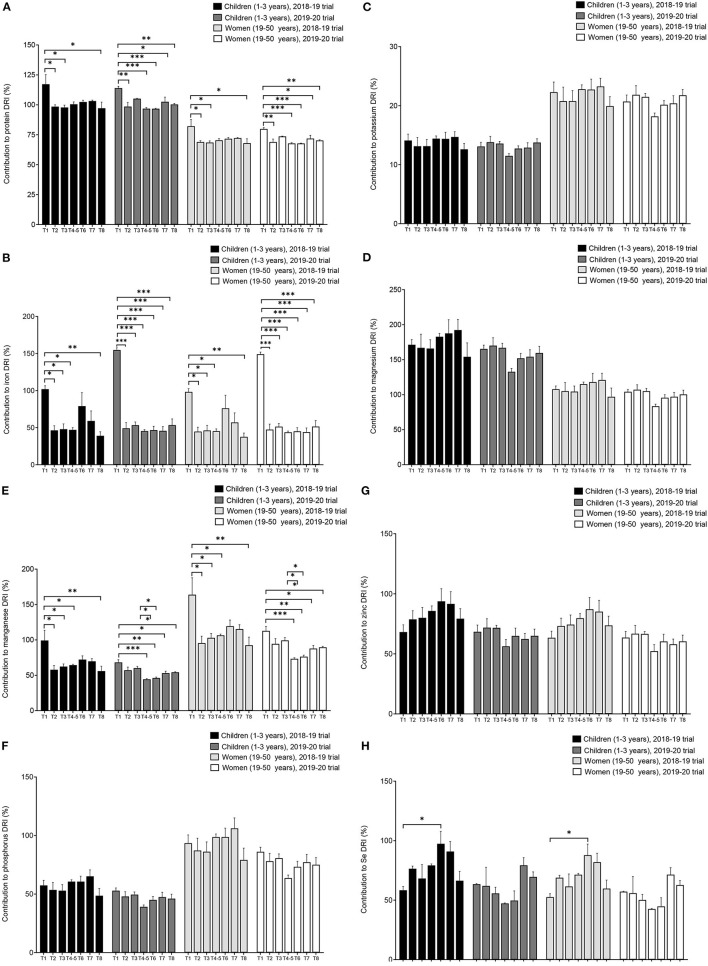
Effect of conservation agriculture on the contribution of maize variety DKC 9089 to the nutrient dietary reference intakes of Malawian children and women. Error bars represent SEM. *, **, and *** mean, significant at 0.05, 0.01, and 0.001, respectively, Tukey's HSD test. Treatments: T1 = conventional agriculture; sole maize. T2 = conservation agriculture (CA); basin sowing; sole maize. T3 = CA; dibble stick sowing; sole maize. T4–5 = CA; dibble stick sowing; maize-cowpea rotation. T6 = CA; dibble stick sowing; maize-pigeon pea intercrop. T7 = CA; dibble stick sowing; maize-cowpea intercrop. T8 = CA; dibble stick sowing; maize-velvet bean intercrop. DRIs, dietary reference intakes.

CA treatments resulted in a small significant reduction of the protein contribution to DRI, an important reduction of the contribution to Fe and Mn needs, no effect was shown on contribution to DRI of K, Mg, and P. A considerable but not significant increase of the contribution to requirements of Zn was observed in the first year of CA trial, and an increase of the contribution to Se needs was obtained with most of the CA treatments. This increase which was significant for treatment T6 (CA; dibble stick sowing; maize-pigeon pea intercrop) in the 2018-19 trial (Figure 9).

Global crop and economic models projected 1–29% increase in cereal price by 2050 due to climate change. This could result in a serious impact on consumers globally through higher food prices, with a high risk on low-income consumers, among which 1–183 million additional people could be at risk of hunger. By increasing the yield, which results in more food supply, climate-smart agricultural practises including CA could help relieve this crisis (28). The positive effect of CA on Se and, to a lesser extent, Zn intakes in Malawi could contribute in reducing the burden of deficiency of these minerals in the population. The small reduction in protein and insignificant effect of CA on K, Mg, and P needs could be reverted by the substantial increase in grain yield by the CA treatments, which will result in the overall higher supply of these nutrients. However, the considerable reduction in Fe supply by CA treatments is a serious concern, especially with the already high prevalence of anaemia in Malawian women and children. In SSA, maize alone can satisfy energy demand but cannot ensure nutrition security, and needs to be complemented by a diversified diet (4, 76, 77). In fact, maize contains a higher amount of carbohydrates and lower content of proteins than legume grains, which are rich sources of essential micronutrients, such as Zn, pro-vitamin A, and Fe (4, 23). Crop diversification in CA systems achieved by intercropping or rotating maize with grain legumes can have a direct impact on diet diversity and nutritional status of the population (35). An overall assessment of the effect of maize-based CA system on nutrients, including the accompanying legume crops will better demonstrate the effect of CA on nutrition security in SSA; for example, higher contents of some nutrients in the legumes could compensate for the low content of other nutrients in maize. The agricultural policy in Malawi has, among others, recommended maize-legume intercrop under CA for improved nutrition outcomes (78).

### Policy Implications in Malawi

It is important to acknowledge that agricultural policies can have consequences on nutrition security. This study has shown that hybrids yield more grain than OPVs. The Malawi National Agriculture Policy (NAP) 2016 (78) reported that agricultural production and productivity have not increased sufficiently over time to match the growing domestic demand and available export opportunities, hence the promotion of hybrid varieties, irrigation, and CA practises. The adoption of drought-resistant hybrid varieties in Malawi is positively influenced by previous season dry spells and access to seed subsidies (79). However, local varieties are still popular among Malawian farm households despite the proliferation of hybrids, owing to their favourable processing and consumption traits, such as taste, storability, poundability, high flour-to-grain ratio, and lower requirements for organic fertilisers (80). Moreover, the cost of hybrid seeds is beyond the reach of many smallholder farmers in Malawi, resulting in over 70% of farmers using recycled seeds (81). The present agricultural input subsidies have increased the uptake of hybrid seeds among smallholder farmers, but there is a dire need to make prices of hybrid seeds accessible to farmers outside the input subsidy program, so as to reduce the proportion of farmers recycling seeds.

In this research study, the observed low nutrient yield of the local variety, which is still grown by a significant number of smallholder farmers, calls for additional policy alternatives to address nutritional outcomes among poor resource farmers. Knowledge of nutrient content of hybrid varieties may further encourage their uptake. Furthermore, the promotion of crop diversification and fortification of food staples should increase nutrient intakes. Presently in Malawi, only sugar is fortified; however, there is a need to envisage fortification of healthy, pro-poor food staples like maize.

The Malawi National Multi-Sector Nutrition Policy (NMSNP) 2018–2022 (82) reported a decrease in anaemia in preschool children from 55 to 28% and called for continuous efforts to address MNDs, such as increasing dietary Fe intake. This study finding on decreased maize grain Fe content under CA is not supporting the policy efforts and needs more collaborative research on alternative CA options for increasing Fe outputs under CA. Crop diversification and maize intercropping involving vegetables rich in Fe, such as Amaranthus species, should be investigated and encouraged under CA production systems. The NMSNP also noted an increased prevalence of cardiovascular diseases mostly due to increased cases of obesity among the population. However, besides addressing obesity challenges, micronutrients play a significant role in reducing the prevalence of cardiovascular diseases (17). There is a need for more awareness of other local foods rich in K, which could be incorporated in CA cropping systems, such as pumpkins and spinach. Zn deficiency is another emerging public health concern highlighted in the NMSNP. This study showed that CA had no significant effect on the Zn yield of maize grain, hence, the need for more research on how grain Zn yield under CA could be increased to meet nutrition policy outcomes. The legumes intercropped with maize in CA systems are known to be particularly rich in Zn, and the overall Zn output of CA should be investigated. Se uptakes and levels of Se markers are low in the Malawian population (14, 16). The present result of increased Se under CA significantly contributes to the NMSNP objectives, and there is a need for more awareness on these nutrition outcomes for increased adoption of CA practises.

In fact, CA adoption in Malawi remains low at 1–2% of cultivated land, and only about 4% of cultivated land is under irrigation (83). The NAP intends to increase by 60% the number of new agricultural technologies under development and being demonstrated to farmers (78). The findings of the current field trial have contributed to show the potential benefits of CA, thus supporting the implementation of this NAP. There is an urgent need for increased public resources towards agriculture research and extension to scale up farm research on CA and generate more evidence on existing research gaps, for increased farmer adoption of the CA practises. The NMSNP aims at ensuring that evidence-based, high-impact nutrition interventions are developed and implemented on scale.

## Conclusion and Perspectives

This study revealed the grain content and yield of essential minerals for three OPVs and five hybrid maize varieties grown at the CARS in Malawi, and how these could contribute to meeting the nutrient requirements of women and children. It also showed the impact of different CA practises on maize grain yield and nutrient content, which resulted in increased yield of proteins and Se, and reduced yield of Fe. Conservation agriculture could help mitigate the negative impact of climate change on maize productivity and food security by increasing the yield and amount of some nutrients available for human consumption. The potential benefits of CA on food and nutrient security of Malawi demonstrated in this study support the policies of the country on agriculture and nutrition that promote high-yielding varieties and CA practises. Nutrient content should help promote CA adoption by smallholder farmers. We recommend that further field trials need to be undertaken in other agroecological regions in Malawi and over more seasons. Furthermore, more studies on the impact of CA on the content and bioavailability of soil minerals, and on their uptake, metabolism, and distribution in the maize plant are needed. Moreover, additional studies are needed on how CA affects maize essential amino acids, anti-nutritional compounds, and other phytochemicals; the response of different maize varieties to CA; and overall nutrient assessment of CA including the legume crops.

## Data Availability Statement

The original contributions presented in the study are included in the article/Supplementary Material, further inquiries can be directed to the corresponding author.

## Author Contributions

IL, MK, CT, AD, YGa, YGo, and CO contributed to study design. YGa, IL, MK, DK, AK, and PK contributed to investigation. YGa, YGo, and CO contributed to data analysis and drafting of the manuscript. CT, AD, YGo, and CO contributed to resources. All the authors contributed to the critical revision of the manuscript and approved the submitted version.

## Funding

This study was supported by the Biotechnology and Biological Sciences Research Council through UK Research and Innovation as part of the Global Challenges Research Fund, AFRICAP programme, grant number BB/P027784/1.

## Conflict of Interest

The authors declare that the research was conducted in the absence of any commercial or financial relationships that could be construed as a potential conflict of interest.

## Publisher's Note

All claims expressed in this article are solely those of the authors and do not necessarily represent those of their affiliated organizations, or those of the publisher, the editors and the reviewers. Any product that may be evaluated in this article, or claim that may be made by its manufacturer, is not guaranteed or endorsed by the publisher.
