# Supplementary material for: Conservation Agriculture Affects Grain and Nutrient Yields of Maize (Zea Mays L.) and Can Impact Food and Nutrition Security in Sub-Saharan Africa
Source: Front Nutr. 2022 Jan 26;8:804663. doi: 10.3389/fnut.2021.804663 (PMC8826957; doi:10.3389/fnut.2021.804663)
Supplement: Supplementary file 1 [file Table_1.docx]

**Supplementary material 1**.

Weather parameters for the 2018-19 and 2019-20 growing seasons at Chitedze Agricultural Research Station, Lilongwe, Malawi.

| **Farming season** | **2018-19** | | | | | | **2019-20** | | | | | |
| --- | --- | --- | --- | --- | --- | --- | --- | --- | --- | --- | --- | --- |
| Months | Dec | Jan | Feb | Mar | Apr | May | Dec | Jan | Feb | Mar | Apr | May |
| Average temperature (°C) | 23.3 | 23.3 | 23.0 | 22.9 | 22.3 | 21.0 | 24.0 | 23.1 | 23.3 | 22.9 | 22.2 | 19.9 |
| Total rainfall (mm) | 170 | 284 | 114 | 184 | 6 | 0 | 224 | 232 | 239 | 56 | 4 | 0 |
| Average relative humidity (%) | 72 | 78 | 77 | 74 | 72 | 67 | 70 | 78 | 80 | 73 | 64 | 61 |
